# Supplementary material for: Musculoskeletal Chronic Graft versus Host Disease—A Rare Complication to Allogeneic Hematopoietic Stem Cell Transplant: A Case-Based Report and Review of the Literature
Source: Curr Oncol. 2022 Nov 3;29(11):8415–30. doi: 10.3390/curroncol29110663 (PMC9689675; doi:10.3390/curroncol29110663)
Supplement: Supplementary file 1 [file curroncol-29-00663-s001.zip › curroncol-1983651-supplementary.pdf]

Supplementals

Musculoskeletal Chronic Graft versus Host Disease – A Rare Complication to Allogeneic Hematopoietic Stem Cell Transplant: A Case-Based Report and Review of the Literature

Alexander Dåtland Kvinge [1,†], Tobias Kvammen [1,†], Hrvoje Miletic [2,3], Laurence Albert Bindoff [4,5] and Håkon Reikvam [1,6,*]

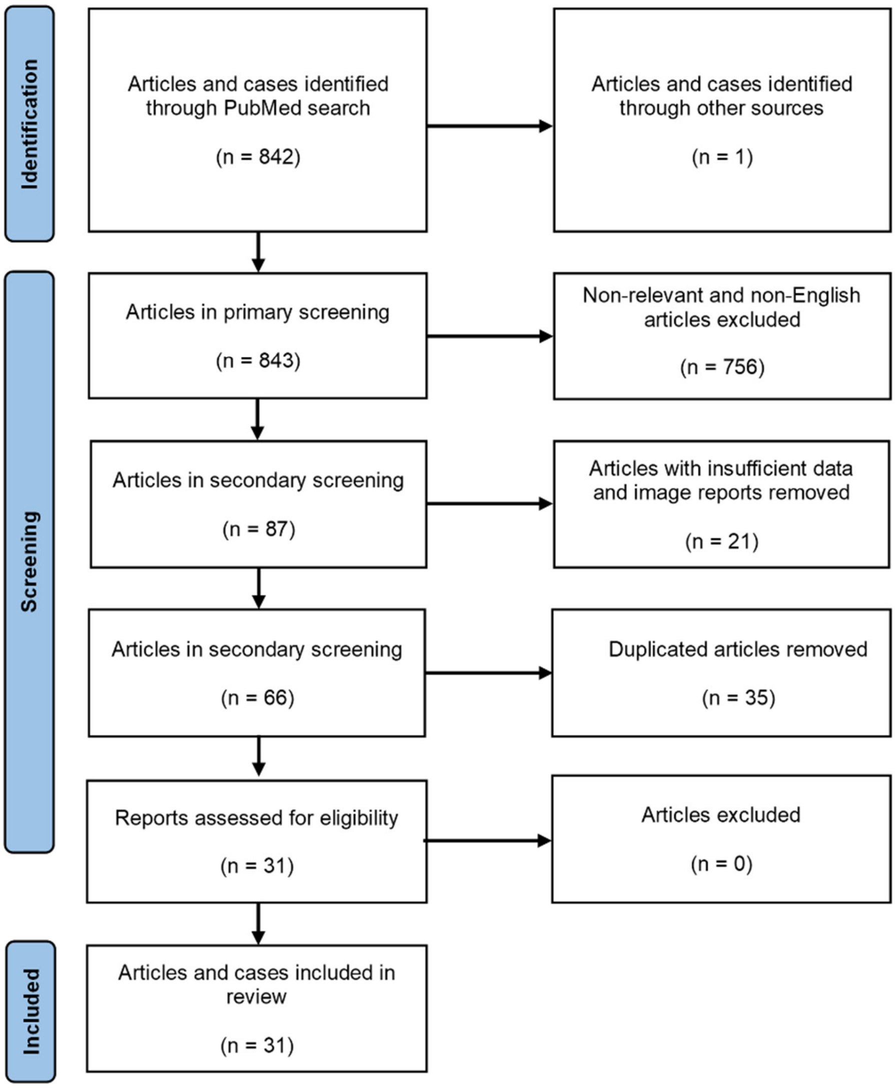

Figure S1. Flowchart demonstrating the selection of the articles used for 161 the systemic review.
